# Extraskeletal myxoid chondrosarcoma of the buttock: a case report and literature review

**DOI:** 10.3389/fonc.2023.1249928

**Published:** 2023-12-13

**Authors:** Xinghua Ji, Jinzheng Wei, Xiaoqiong Li, Wei Zhang, Zejun Xing

**Affiliations:** ^1^ Department of Orthopedics, Shanxi Bethune Hospital, Shanxi Academy of Medical Sciences, Tongji Shanxi Hospital, Third Hospital of Shanxi Medical University, Taiyuan, China; ^2^ First Clinical Medical College, Shanxi Medical University, Taiyuan, China; ^3^ Department of Magnetic Resonance Imaging, Taiyuan Central Hospital of Shanxi Medical University, Taiyuan, China; ^4^ Economics College of Hebei Geo University, Shijiazhuang, Hubei, China

**Keywords:** extraosseous myxoid chondrosarcoma, soft tissue sarcoma, radiography, pathology, case report

## Abstract

**Background:**

Extraosseous myxoid chondrosarcoma (EMC) is extremely rare, and the case we report is of a particular site with partial bone destruction.

**Case presentation:**

This case report can further strengthen the understanding of EMC and guide clinical treatment. The patient presented with a right buttock mass that was present for 1 year and that had gradually enlarged with tenderness for 6 months. The diagnosis was EMC. The interventions included puncture biopsy, surgical resection, and postoperative chemotherapy. The tumor was resected extensively, and the postoperative recovery was satisfactory. There was no recurrence or metastasis during the follow-up for 18-month.

**Case presentation:**

The case we reported occurred in the pelvic cavity, which has not been previously reported in the literature, and there was partial bone destruction. Complete resection of the tumor was performed, and a satisfactory prognosis was achieved.

## Introduction

EMC accounts for almost 3% of all soft tissue sarcomas (1). It is a rare tumor with an indolent course and a high propensity for local recurrence and metastasis (2). Because EMC is very rare, the clinical manifestations are not characteristic, and early diagnosis before surgery is difficult. This article reports a case of EMC with bone destruction in the buttocks and reviews relevant literature to improve the understanding of the disease and to provide valuable information for clinical diagnosis and treatment.

## Case description

Age and sex: 53-year-old female

Chief complaints: the patient presented with a mass in the buttock that had been growing in size since 2019.

History of present illness: The right buttock mass was present for 1 year and was gradually increasing in size. The clinical manifestations were symptoms of rectum and uterus compression, with discomfort, dragging sensation, and distension in the lower abdomen, and the mass inhibited defecation.

History of past illness: The patient had hypertension for 10 years, with stable blood pressure control.

Family history: None of her family members were diagnosed with carcinoma.

Physical examination: A fixed mass was present in the right buttock, with no skin changes or ulceration; the local boundary was still clear; and the patient had local tenderness. The tumor firmness and mobility were poor.

The laboratory testing showed the following: Serum red blood cell count: 3.5*10^12^/L; serum white blood cell count: 5.7*10^9^/L (neutrophil percentage: 65%, lymphocyte percentage 23.7%); hemoglobin concentration: 113 g/L; hematocrit: 0.330 L/L; serum alanine aminotransferase concentration: 23.0 IU/L; serum aspartate aminotransferase: 20.0 IU/L; serum albumin concentration: 39.8 g/L; serum triglyceride concentration: 2.23 mmol/L; serum urea concentration: 5.0 mmol/L; serum creatinine concentration: 73.2 μmol/L; serum uric acid concentration: 320.3 μmol/L; alpha-fetoprotein: 1.5 ng/ml; carcinoembryonic antigen: 0.9 ng/ml; carbohydrate antigen199: 6.9 U/ml, carbohydrate antigen125: 2.2 U/ml, carbohydrate antigen153: 4.3 U/ml, and carbohydrate antigen242: 2.9.

**Figure 1:**
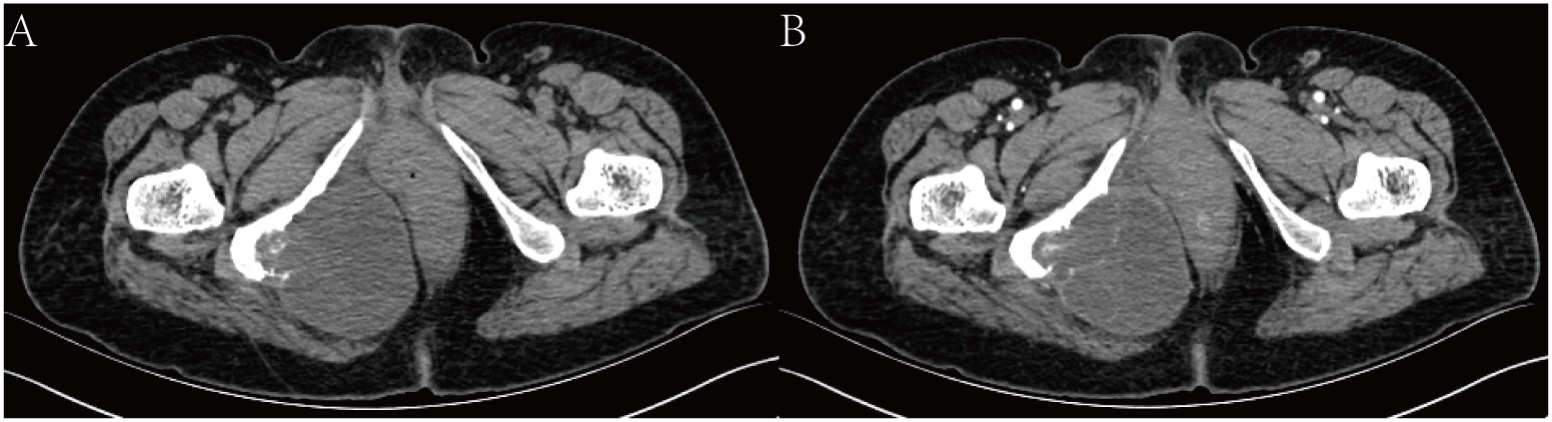
CT scan. **(A)** shows a mass of cystic low-density shadow on the right side of the ischiorectal fossa. **(B)** shows enhanced scanning of the cyst wall, and segmentations showed mild enhancement.

Imaging examination: Computed tomography (CT) showed that there was a mass of cystic low-density shadow on the right side of the ischiorectal fossa, and the mass was approximately 6.95 cm×7.79 cm, with segmentations visible inside (**Figure 1A**). Enhanced scanning of the cyst wall and segmentations showed mild enhancement and visible right internal iliac artery branches supplying blood to the tumor. There was adjacent ischium bone destruction, and the adjacent rectum and vagina were pushed to the left (**Figure 1B**).

**Figure 2:**
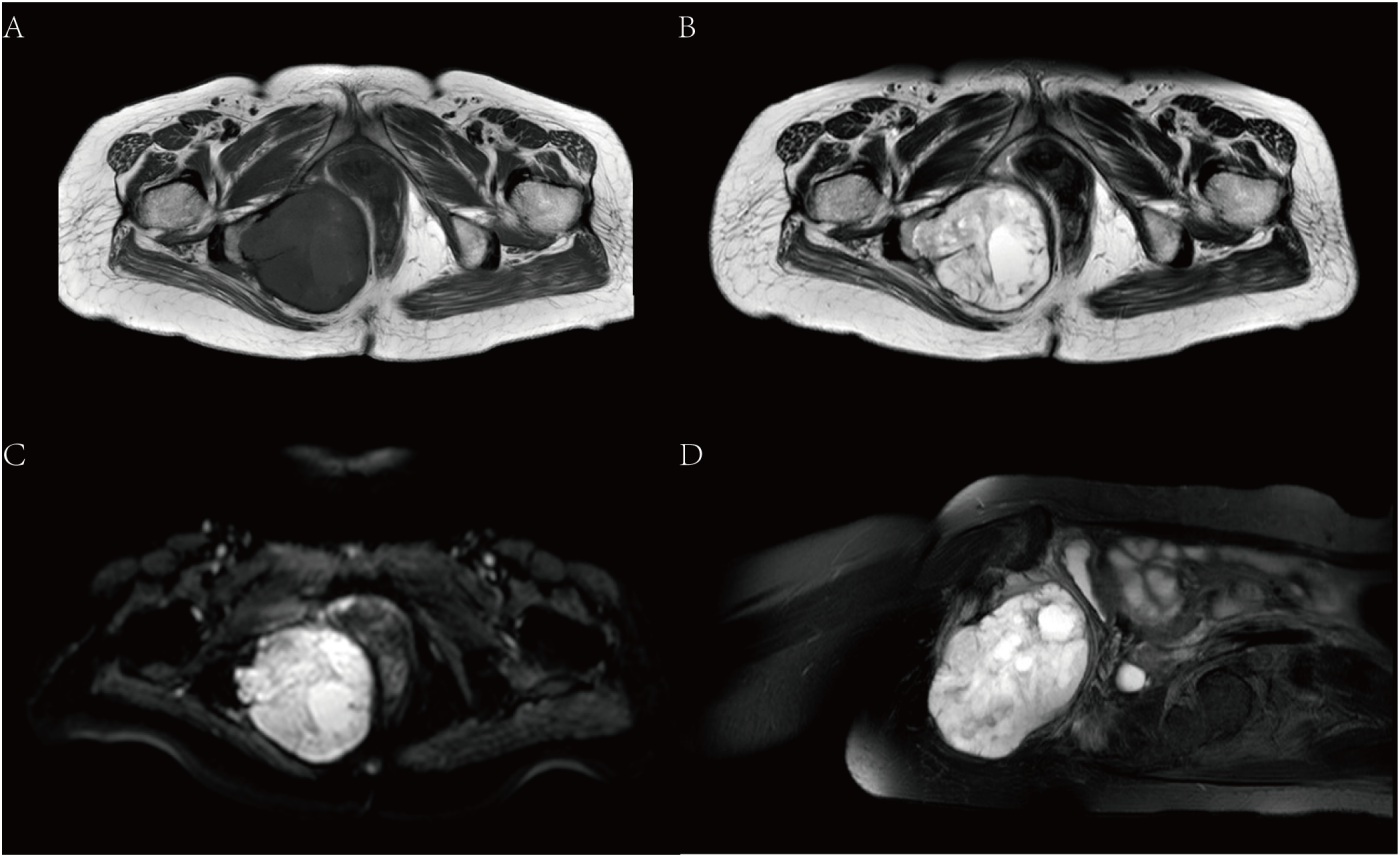
MRI. **(A)** is T1, and **(B)** is T2. **(C)** shows DWI images with high signals. **(D)** shows the sagittal position of the T2 fat suppression sequence.

Magnetic resonance imaging (MRI) showed that there was a mass of mixed T1 and T2 signal shadows in the right ischiorectal fossa. The main body of the lesion showed equal T1 and long T2 signal shadows, and small patches of shorter T1 and equal T2 signal shadows were visible (**Figures 2A, B**). Diffusion-weighted imaging (DWI) and apparent diffusion coefficient (ADC) images showed a high signal (**Figure 2C**). The obturator internal muscle was unclear since it was involved in the lesion. The adjacent tissues were pushed to the left. The tumor was approximately 8.55 cm×7.59 cm×8.32 cm. The adjacent soft tissues showed a grid-like high signal in the fat suppression sequence (**Figure 2D**).

Genetic testing: none

Final diagnosis: Immunohistochemical staining of the tumor showed positive staining for S100 and Vimentin, suspicious positivity for mesothelial cells, and negative staining for cytokeratin AE1/AE3, EMA, CK19, E-cadherin, and CK5/6. The Ki67 labeling index reached approximately 40% in some areas.

**Figure 3:**
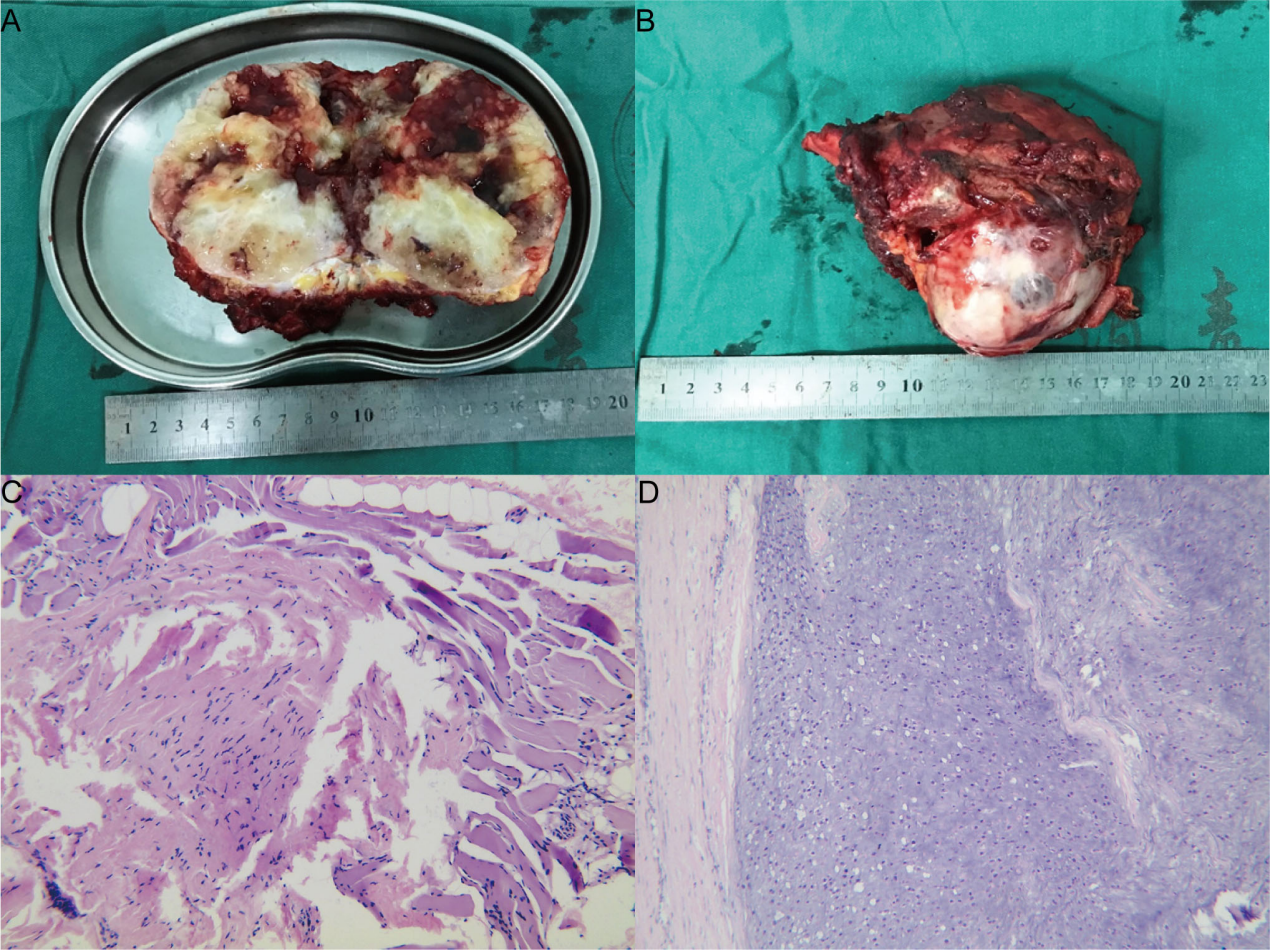
Pathology. **(A)** is a general observation, and **(B)** is a section observation. **(C)** shows the needle biopsy, and some spindle cells were visible. **(D)** is HE×100.

The tumor stroma showed mucinous denaturation, the tumor cells were polygonal and epithelioid, the nuclei were slightly atypia, grid-like calcification, and reactive bone formation were found within local lesions, and the bone tissues were invaded by the lesion. All the changes pointed to the diagnosis of EMC (**Figures 3C, D**).

Treatment: Before the surgery, the tumor supplying artery was embolized through intervention. Subsequently, a needle biopsy of the tumor was performed, and the results showed that some spindle cells were visible (**Figures 3C, D**). We performed a resection of the tumor with an extended range of excision. During the surgery, we made a curved incision along the posterior midline of the sacrum at the level of S4, extending towards the right buttock tumor area. The incision was approximately 30cm long. The layers of skin, subcutaneous tissue, and deep fascia were sequentially dissected. Blunt dissection was performed from the tailbone area towards the right buttock to expose the tumor, while taking care to protect the gluteus maximus, gluteus medius, and gluteus minimus muscles. The proximal portion of the tailbone was removed first, revealing that the tumor extended anteriorly from the right buttock to the pelvic cavity, with an intact capsule. After thorough exposure of the posterior aspect of the tumor, the tumor was bluntly dissected along its margins. Exploration revealed the tumor closely related to the ischial tuberosity with significant involvement of the bone. Partial resection of the ischial tuberosity was performed to further separate the tumor deeply into the pelvic cavity. Exploration indicated that the tumor was closely related to the right pubic branch with bone involvement. To protect the surrounding tissues, partial resection of the pubic branch was performed using a saw. At this point, exploration confirmed complete separation of the tumor from the surrounding tissues, and the tumor was completely excised (**Figures 3A, B**). No infiltration was observed at the tumor margin. After surgery, the patient received three rounds of chemotherapy (Anlotinib, 10mg, QD, 14days) after surgery.

**Figure 4:**
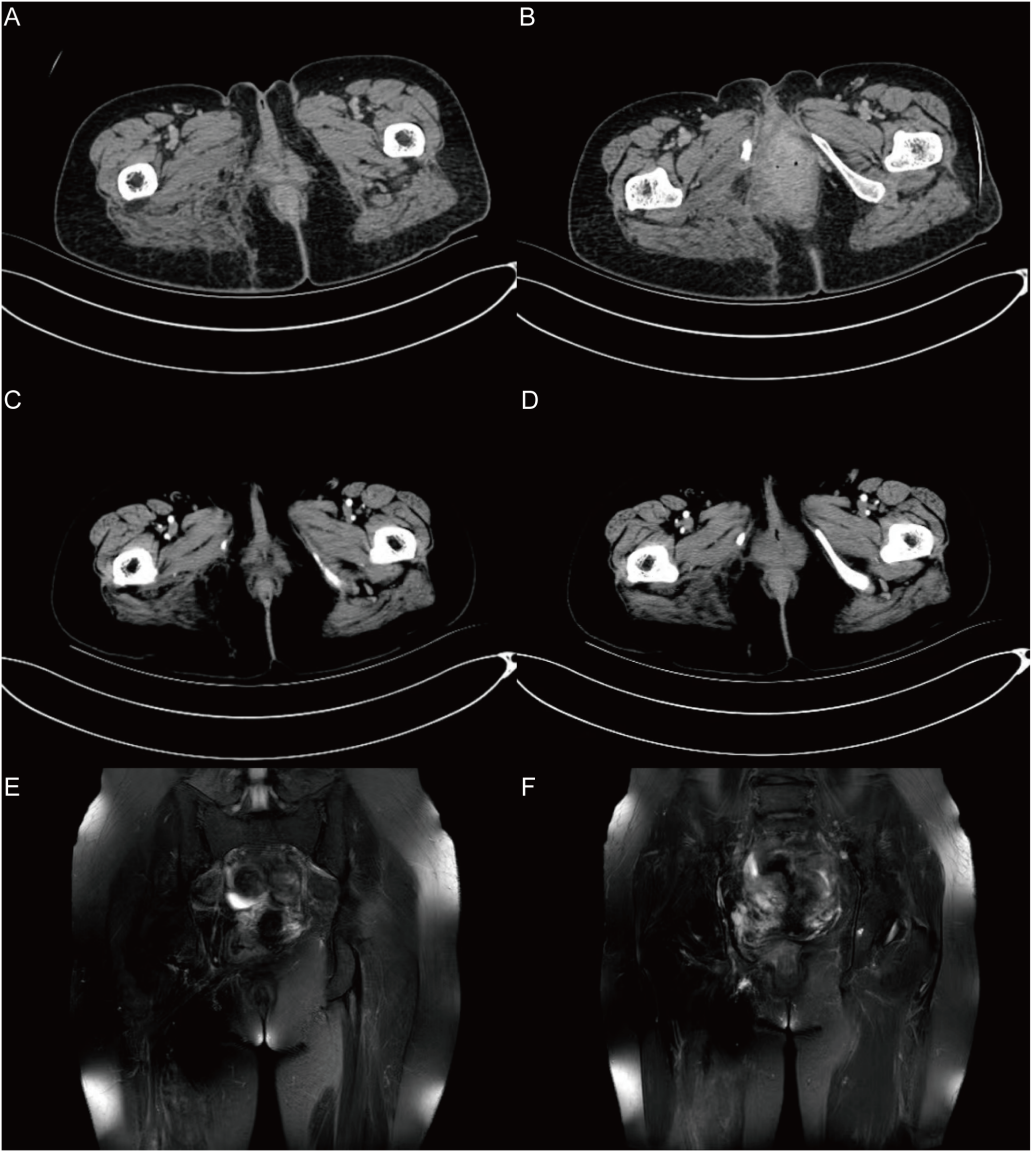
The follow-up results in 18 months after surgery. **(A)**, **(B)** show the CT results after 1 month. **(C)**, **(D)** show the CT results after 6 months. **(E)**, **(F)** show the MRI results after 18 months. In the imaging examination results during the 18-month follow-up after surgery, no recurrence or metastasis was observed.

Outcome and follow-up: The patient recovered well after the operation, the patient’s limb function was normal, and there was no recurrence or metastasis during the 18-month follow-up after the operation (**Figure 4**).

## Discussion

Primary EMC is a mesenchymal tumor with an undetermined differentiation direction (3). Due to its rare incidence, only 5 cases have been reported in the literature to date. We also collected information on the 5 previously reported cases of primary buttock EMC (shown in **Table 1**) (4–8) to perform a systematic review of this tumor type and discussing the epidemiological and radiographic features, diagnosis, treatment strategies, and prognosis of this rare disease. The case presented herein is of a 52-year-old female patient with primary buttock EMC with bone destruction. To the best of our knowledge, this is the first case of primary buttock EMC with bone destruction.

**Table 1:** Published and present cases of primary buttock EMC.

Reference	Gender/ages	Size of tumor (cm)	Bone involvement	Treatment	Recurrence	Metastasis	Follow up
Theodore et al (4), 1994	M/61	17×11	N	GTA,PC	N	N	2y
Goran Stenman et al (5), 1995	M/71	15×12×8	N	GTA	N	N	2.5y
M.Harris, et al (6), 2000	M/54	9×8×5	N	FNA,GTA	N	N	NA
Joseph, et al (7), 2007	M/68	2	NA	FNA,GTA	NA	NA	NA
Zhang, et al (8), 2014	M/47	8×6×6	N	GTA	N	N	NA

GTR, Gross total resection; PC, Postoperative chemotherapy; FNA, Fine-needle aspiration biopsy; N, No; NA, Not available.

EMC manifests as a solitary soft tissue mass, which easily recurs after surgery and can be transferred. Usually, it does not invade bone tissue. It often occurs in the soft tissues of the proximal limbs and deep tissues of middle-aged people. The incidence in males and females is approximately 2:1. EMC is a relatively rare neoplasm with no specific findings in clinical practice. Patients commonly present with nonspecific symptoms, including tenderness and the detection of a palpable mass (9). The most common manifestation of EMC is an enlarging soft tissue mass, and some lesions are accompanied by pain and tenderness or may restrict the range of motion. This patient, a 53-year-old female, presented with a gradually enlarged mass on the right hip, with local tenderness and involvement of the pelvis; CT scanning showed that the tumor had invaded the ischial tuberosity, which is extremely rare in EMC. The tumor found in this bone is unusual since only 16 cases have been reported in the literature.

There were no specific CT findings for primitive EMC (10, 11), mostly shown as heterogeneous tumors in soft tissue. The existence of EMC is also characterized by tumors with low density (mostly 20-40 HU) on CT images and mild or no enhancement on enhanced CT images (10, 11). The average CT value of the tumor, in this case, was 20 HU, and the segmentation was slightly enhanced, which was consistent with the findings in the literature. Calcification is rarely found on the CT images of EMC. Zhang et al. retrospectively analyzed 13 EMC patients, 3 of whom showed calcification (12). EMC is characterized by the presence of a cartilage matrix on MR images. The mass showed a lobulated appearance with iso-/hypo-intensity to muscle on T1WI and hyperintensity on T2WI, and it was usually segmented into multiple lobules by multiple septa with hypointensity. The signal intensity was higher in the myxoid areas than in the solid areas, and the calcified areas appeared hypointense on T2WI. In contrast-enhanced images, peripheral, septal, or heterogeneous enhancement patterns were observed (13).

There was a large difference in the tumor sizes by the naked eye. The cut surfaces are greyish-white or grey-brown. The tumors are composed of multiple jelly-like tumor nodules. The nodules are separated by fibrous tissue, and some of them have cystic denaturation, hemorrhage, and necrosis. It can be seen under the microscope that the tumor nodules are composed of various interconnected eosinophilic spindles and oval and small round cells, and these cells are separated by a mucus-like and cartilage-like matrix in a mesh or lace shape. Positive S-100 and lack of INI-1 expression are helpful for diagnosis (14). The positivity of GFAP has been reported. Under electron microscopy, the presence of parallel microtubules and abundant REG are quite characteristic but not specific (15, 16). Certain studies have shown that they may also be positive for Leu-7 and epithelial membrane antigens. Uniformly, they are negative for SMA, and desmin (8, 17, 18). Under the electron microscope, the characteristic microtubules in the pool are arranged in bundles or parallel. In some cases, intracellular neuroendocrine granules can be seen. Therefore, EMC can express the neuroendocrine markers Syn and NSE. INSM1 could be a potential marker for the diagnosis of EMC when molecular genetic access is limited (19).

Complete surgical resection is the main treatment method for EMC. The early literature reported that EMC was not sensitive to radiotherapy and chemotherapy. With the deepening understanding of EMC, radiotherapy and chemotherapy have been found to show a certain value. Radiotherapy can reduce postoperative complications from 41% to 20% (20). Receiving external beam radiotherapy (EBRT) is associated with a cancer-specific survival benefit in localized EMC. Aggressive local therapy, including EBRT, should be considered in these patients (21). The studies in the past were aimed at improving disease control by surgical resection or radiotherapy, with some reported benefits from the tyrosine kinase inhibitor sunitinib malate in the metastatic setting (21–24). Recent research has shown that pazopanib has antitumor action with clinical value in patients with progressive and advanced EMC, which could be considered a suitable option after these patients fail to respond to first-line anthracycline-based chemotherapy (25). This provides new ideas for the treatment of EMC. Oliveira et al. (26) believe that the indicators of poor prognosis include older age, tumor diameter > 10 cm, tumor metastasis, histological anaplastic area, mitotic figures > 2/10 HPF, and Ki-67 proliferation index ≥ 10%. In this case, the Ki-67 proliferation index was ≥40%. The patient received three rounds of chemotherapy after surgery. No recurrence or metastasis occurred during the 18-month follow-up after the operation.

In conclusion, EMC is a rare malignant tumor. The case we reported occurred in the pelvic cavity, which has not been reported in the literature, and there was partial bone destruction. Complete resection of the tumor was performed, and a satisfactory prognosis was achieved. The report of this case provides theoretical support for the clinical diagnosis and treatment of EMC.

## Data availability statement

The raw data supporting the conclusions of this article will be made available by the authors, without undue reservation.

## Ethics statement

Written informed consent was obtained from the participant/patients(s) for the publication of this case report.

## Author contributions

XJ: data acquisition, literature search, manuscript preparation, and medical management. JW: data acquisition, literature search, and manuscript preparation. XL: pathological interpretation, manuscript preparation, and editing. WZ: data acquisition and manuscript preparation. ZX: data acquisition, literature search, and manuscript preparation. All authors read and approved the final manuscript. All authors contributed to the article and approved the submitted version.
